# Multi-Image Encryption Method via Computational Integral Imaging Algorithm

**DOI:** 10.3390/e24070996

**Published:** 2022-07-18

**Authors:** Xiaowu Li, Chuying Yu, Junfeng Guo

**Affiliations:** 1 The Second Affiliated Hospital of Shantou University Medical College, Shantou 515000, China; lxw_841121@163.com; 2 School of Physics and Electronic Engineering, Hanshan Normal University, Chaozhou 521041, China; 3 School of Electronics and Information Engineering, Sichuan University, Chengdu 610065, China

**Keywords:** multi-image encryption, computational integral imaging, DNA-chaos algorithm

## Abstract

Under the framework of computational integral imaging, a multi-image encryption scheme based on the DNA-chaos algorithm is proposed. In this scheme, multiple images are merged to one image by a computational integral imaging algorithm, which significantly improves the efficiency of image encryption. Meanwhile, the computational integral imaging algorithm can merge images at different depth distances, thereby the different depth distances of multiple images can also be used as keys to increase the security of the encryption method. In addition, the high randomness of the chaos algorithm is combined to address the outline effect caused by the DNA encryption algorithm. We have experimentally verified the proposed multi-image encryption scheme. The entropy value of the encrypted image is 7.6227, whereas the entropy value of the merge image with two input images is 3.2886, which greatly reduces the relevance of the image. The simulation results also confirm that the proposed encryption scheme has high key security and can protect against various attacks.

## 1. Introduction

As a basic way of carrying data, the importance of images in the information industry is self-evident. However, there is no doubt that this will raise a lot of privacy concerns if the image information owned by an individual or team is accessed by others. Image encryption is a proven means of solving image security problems [1,2,3,4,5,6]. Encrypted images lack intuitive information about the original image, in other words, the thief cannot obtain any valuable information from the encrypted image, thereby achieving the privacy protection of the image owner. At present, researchers have proposed many methods of image encryption, and optical encryption has attracted much attention in the study of image encryption because of its unique multi-dimensional capabilities, high parallelism and high-speed processing power [7,8,9,10,11,12].

Since Javidi and Refregier proposed the classic optical Dual Random Phase Encoding (DRPE) system in 1995 [13], optical encryption technology began to enter a period of rapid development. Researchers have found that the encryption system based on DRPE technology has some security problems due to its own linear factors [14]; it is vulnerable to selective plaintext attacks. In order to enhance the security of the encryption system, researchers have proposed a series of feasible optical encryption improvement schemes based on DRPE. The improvements are mainly made from the following four aspects: (1) Expansion of optical transformations; (2) Pre-process according to the characteristics of the encrypted image and the purpose of encryption; (3) Improvements of the random phase mask; (4) Non-linear operation. On this basis, more encryption algorithms have been proposed [15,16,17,18,19,20]. It is worth noting that these proposed methods of encrypting objects are all for a single image. Compared with single image encryption, multi-image encryption can process multiple images at a time, which can greatly improve encryption efficiency on the basis of ensuring encryption security.

Computational Integral Imaging (CII), as a well-performing optical imaging system, can achieve a full-color, wide-angle 3D light field display [21,22,23,24,25,26,27]. Integral imaging technology can record image information from multiple perspectives of a scene, which can provide more robustness in the recovery process of image encryption. Researchers have proposed many image encryption methods based on the CII framework, and confirmed that these algorithms have strong robustness [28,29,30]. In addition, computational integral imaging technology can also record scene information at different depths; at the same time [31,32,33], it is possible to achieve the synthesis of multiple images, which provides a new idea for multi-image encryption.

DNA algorithm with vast parallelism, large-scale storage and extraordinary information density is often applied in the study of data encoding, and some DNA-based encoding algorithms were proposed and showed a better performance [34,35,36,37,38,39]. The proposed DNA-based encryption algorithms have two main ideas—one is to explore the impact of different DNA rules on encryption performance, and the other is to improve the performance of DNA encryption by combining other encryption algorithms [40]. There will be an outline effect when DNA encryption algorithm is used, which causes the saliency boundaries of the original image to be seen from the encrypted image clearly. The chaos system possesses a variety of characteristics, such as strong confidentiality, good randomness, a large amount of keys and so on [41,42,43]. In addition, recently, elliptic curves-based image encryption schemes have been considered an alternative to the chaos-based schemes [44,45,46,47]. Therefore, they are widely used in combination with DNA algorithms to improve the performance of encryption systems.

In this paper, a multi-image encryption scheme based on CII using a DNA-chaos encryption algorithm is proposed. Two or more images are merged in different depths using the CII algorithm, which will obtain an Element Image Array (EIA) image, and then the EIA image is encrypted by the DNA-chaos encryption algorithm. Finally, the decrypted image can be reconstructed at different depths to restore the original image. Based on the high security of the DNA-chaos encryption algorithm, EIA data recorded by computational integral imaging technology to ensure the strong robustness and the multi-image merge scheme of different depths further improves the key space by using depth information as the key.

The paper is arranged as follows. In Section 2, we briefly introduce a theoretical analysis of our method, including the CII pickup process, DNA sequence operations, chaos theory, the CII Reconstruction (CIIR) algorithm and entropy analysis theory. A multi-image encryption scheme is proposed in Section 3. In Section 4, we analyze the performance of the proposed multi-image encryption scheme in terms of key security, statistical results, robustness and time analysis. The conclusions reached in this article are presented in Section 5.

## 2. Previous Theoretical Analysis

### 2.1. Pickup Original Scene by CII

CII [22] is an advanced optical imaging solution, which is the most promising commercial 3D display technology, and has important research significance in the field of 3D image processing. The modulation of optical information from CII mainly includes two processes—one is the pickup of the original scene, which can obtain the EIA, and the other is the reconstruction of the original scene through EIA. In the pickup of the original scene, an EIA is recorded, and the EIA contains a lot of redundant information about the scene, which can improve the robustness of the encryption scheme for image encryption.

**Figure 1:**
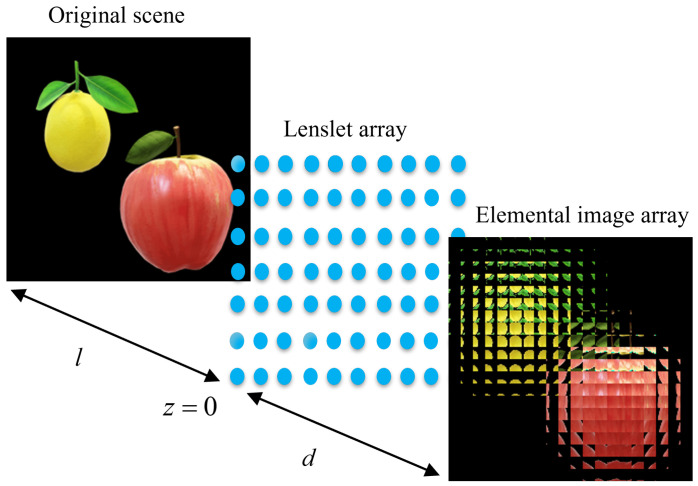
The pickup of the original scene by CII.

Figure 1 shows the pickup of the original scene by CII algorithm. The original scene is recorded by the sensor as EIA through a lenslet array, and the EIA contains many EIs; each EI represents the encrypted information converted by part of the original scene. Each EI is calculated by [22]:

(1) $$E\left(x,y,z\right)=P\left(-\frac{xd}{l}+i\phi ,-\frac{yd}{l}+j\phi ,z\right),$$

where *x*, *y* and *z* represent the spatial coordinates of the lenslet array, and the size of the lenslet array determines the number of EIs. ϕ represents the size of lens, and the distance from image to lenslet array is *l*.

### 2.2. DNA Sequence Operations

A Deoxyribo Nucleic Acid (DNA) sequence consists of four different basic nucleotides: adenine (A), guanine (G), thymine (T) and cytosine (C). These four nucleotides can be combined to form a long sequence, and T is paired with A,G is paired with C. We will obtain 24 different encoding schemes if A,C,G and T are encoded as binary numbers with two bits, respectively, but only eight encoding schemes suit the Watson–Crick rule, and they are shown in Table 1. Assuming that A-10, T-01, C-11, G-00, such as the binary sequence 10110100, the DNA sequence can be written as ACTG.

DNA computing has received a lot of attention from researchers, so it has developed rapidly. Some researchers have proposed certain algebraic operations for DNA sequences, such as addition operations, subtraction operations and Ex-OR operations. Corresponding to the eight DNA coding schemes there are also eight DNA addition, subtraction and Ex-OR operations. Table 2 lists one of the arithmetic rules which, according to DNA encoding rule one, are listed in Table 1 [34].

For DNA recovery operations, the corresponding DNA sequences must meet the conditions specified by [36]:

(2) $$\left\{\begin{array}{c} Y_{B}\neq C_{p}\left(Y_{B}\right)\neq C_{p}\left(C_{p}\left(Y_{B}\right)\right)\neq C_{p}\left(C_{p}\left(C_{p}\left(Y_{B}\right)\right)\right)\\ Y_{B}=C_{p}\left(C_{p}\left(C_{p}\left(C_{p}\left(Y_{B}\right)\right)\right)\right) \end{array}\right.,$$

where $Y_{B}$ denotes one of the four different basic nucleotides, and $C_{p}\left(Y_{B}\right)$ is the base pair of $Y_{B}$.

### 2.3. Chaos Theory

Chaos as a nonlinear dynamic process that is highly sensitive to initial states and is unpredictable becomes a natural physical code. It widely applies in the fields of cryptography, random number generation, confidential communication and image encryption. In this paper, two chaos functions containing SLMM and a logistic map are selected to improve the performance of DNA encryption.

2D-SLMM is defined as [48]:

(3) $$\left\{\begin{array}{c} X(n+1)=\alpha (\sin(\pi Y(n))+\beta )X(n)(1-X(n))\\ Y(n+1)=\alpha (\sin(\pi X(n+1))+\beta )Y(n)(1-Y(n)) \end{array}\right.,$$

where α and β are control parameters, and 0≤α≤1, 0≤β≤3. It should be noted that if we want SLMM to work in a chaotic state, the β should close to 3 [48].

There will be a 1D chaos function to describe the logistic map, which is defined as [49]:

(4) x(n+1)=γx(n)(1−x(n)),

where γ∈[0,4] is the logistic map parameter, and $x_{n}\in \left(0,1\right)$. Only when 3.5699456≤γ≤4, does the logistic map exhibit a state of chaos [49].

### 2.4. CIIR Algorithm

In the multi-image encryption scheme we proposed, the CIIR algorithm is used to recover different image scenes. However, traditional CIIR algorithms easily cause some pixels to coincide, resulting in a decrease in the intelligibility quality of the recovered scene. To improve the effects of pixel coincidence, we apply a modified reconstruction algorithm [50] so that every reconstructed scene pixel can be calculated. The calculation of the original scene uses the following formula:

(5) $$Y_{R}\left(x,y,z\right)=\frac{1}{T_{z}}\sum_{i=0}^{M-1}\sum_{j=0}^{N-1}E_{i,j}\left(x-i\frac{M\times p}{m\times u},j\frac{N\times p}{n\times u}\right),$$

where $T_{z}$ denotes the number of overlaps at the reconstruction distance *z*, *M* and *N* determine the number of EIs, *m* and *n* represent the size of the imaging sensor, *p* represents the pitch between each pinhole, and *u* is the magnification parameter.

### 2.5. Entropy Analysis Theory

To illustrate the performance of our proposed encryption scheme quantitatively, we introduce an entropy analysis method. Image entropy describes the average amount of information in an image, representing the aggregation characteristics of the image pixel distribution [51]. For image encryption, the original image contains more spatial features, the pixel distribution is more dispersed, the entropy value is small. While the encrypted image should contain the original image information as less as possible, the pixel distribution is relatively concentrated, so the entropy value is larger than in the original image. Therefore, the size of the entropy value can be analyzed to judge the performance of the encryption scheme.

The entropy of image *I* can be obtained by the following formula:

(6) $$H\left(I\right)=-\sum_{i=1}^{n}P\left(a_{i}\right)\cdot \log_{2}P\left(a_{i}\right),$$

where $p(a_{i})$ denotes the probability of occurrence of pixel with value of $a_{i}$ in image *I* with $0\leq p(a_{i})\leq 1$ and $\sum_{i=1}^{p}\left(a_{i}\right)=1$.

## 3. Multi-Image Encryption Scheme Based on CII

### 3.1. Framework of Multi-Image Encryption Scheme

**Figure 2:**
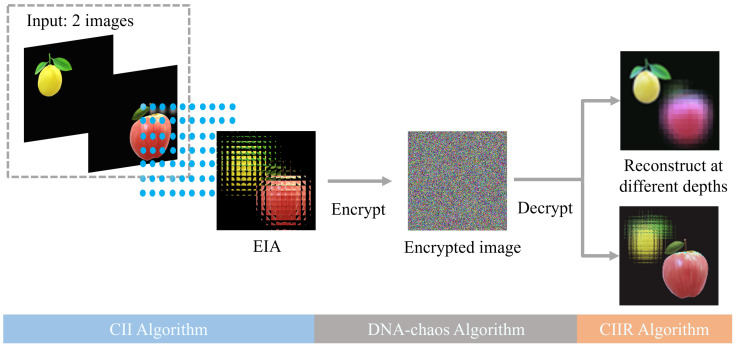
Framework of multi-image encryption scheme.

In this paper, a multi-image encryption scheme is proposed based the principle of CII, and the overall framework of the scheme is shown in Figure 2.

In the multi-image encryption scheme we proposed, the input can be two or more images (two images are shown in Figure 2, and the following is also described in two images). Firstly, the two input images are placed on different position planes, then it is recorded on an EIA by a microlens array. The EIA merges information from two original images. It is worth noting that the different distances between two images and the microlens array can be used as keys. After that, the EIA is encrypted by the DNA-chaos encryption. Finally, the CIIR is used to reconstruct different original images with different depths. The detailed encryption and decryption procedure is described in Section 3.2.

### 3.2. Encryption and Decryption Procedure

**Figure 3:**
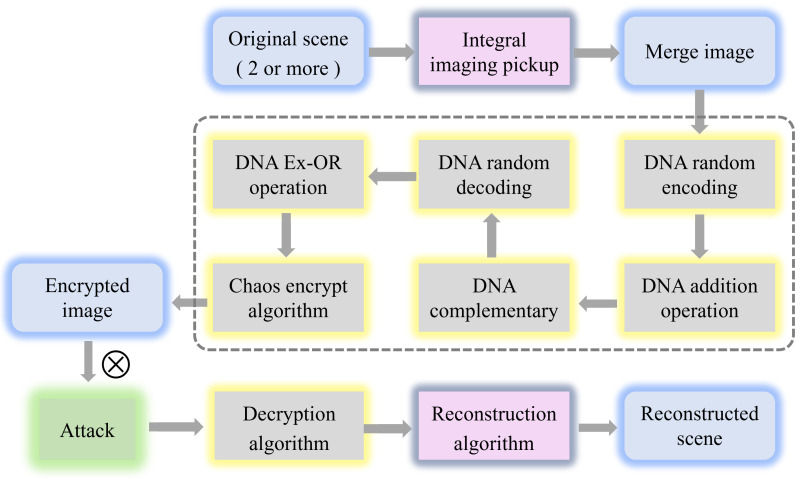
The encryption procedure of multi-image encryption scheme.

**Figure 4:**
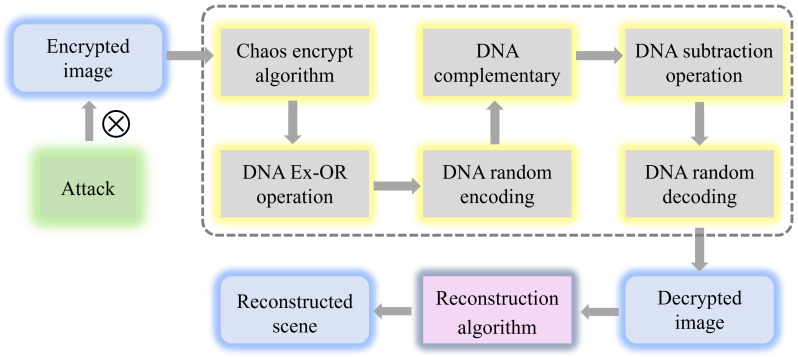
The decryption procedure of multi-image encryption scheme.

Figure 3 shows the detailed steps of the encryption algorithm we proposed. It is worth noting that we introduce the chaos algorithm to solve the outline effect caused by the DNA algorithm. The proposed multi-image encryption scheme is a symmetric process, so the decryption process of the image can be achieved by reversing the encryption process. The steps of the decryption process are shown in Figure 4, we only introduce the encryption procedure of the multi-image encryption scheme in detail.

The encryption procedure of the multi-image encryption scheme is introduced as follows:

**Step 1**: Convert the original scene into the form of merge image f(i,j) with size M×N using the integral imaging pickup algorithm.

**Step 2:** Generate two high-quality pseudo-random sequences $M_{1}\left(i,j\right)$ and $M_{2}\left(i,j\right)$ with size M×N by cellular automata with two different initial states.

**Step 3:** Decompose the merge image f(i,j) to three binary matrices $R_{1}\left(i,j\right)$, $G_{1}\left(i,j\right)$ and $B_{1}\left(i,j\right)$ with the size of M×N. Then transform the three binary matrices into three DNA sequence matrices $R_{2}\left(i,j\right)$, $G_{2}\left(i,j\right)$ and $B_{2}\left(i,j\right)$ with the size of M×4N based on the DNA coding rules defined in Table 1 and the random encoding sequence En_M(i,j) generated from pseudo-random sequences $M_{1}\left(i,j\right)$. The random coding sequence En_M(i,j) can be obtained by:

(7) $$En\_M\left(i,j\right)=floor\left(\;\mathrm{mod}\;\left(M_{1}\left(i,j\right),8\right)\right)+1.$$

**Step 4**: Perform the diffusion operations by DNA addition to get three DNA diffused matrices $R_{3}\left(i,j\right)$, $G_{3}\left(i,j\right)$ and $B_{3}\left(i,j\right)$ with the size of M×4N.

**Step 5:** Select the rule from four complementary rules according to pseudo-random sequence $M_{3}\left(i,j\right)$. Based on $M_{3}\left(i,j\right)$ and the selected complementary rule, perform the DNA complementary operation on DNA diffused matrices and obtain three DNA complementary matrices $R_{3}^{\prime }\left(i,j\right)$, $G_{3}^{\prime }\left(i,j\right)$ and $B_{3}^{\prime }\left(i,j\right)$. The pseudo-random sequence $M_{3}\left(i,j\right)$ is described as:

(8) $$M_{3}\left(i,j\right)=M_{1}\left(i,j\right)⊕M_{2}\left(i,j\right).$$

**Step 6:** Decode three DNA matrices $R_{3}^{\prime }\left(i,j\right)$, $G_{3}^{\prime }\left(i,j\right)$ and $B_{3}^{\prime }\left(i,j\right)$ using DNA random decoding sequence De_M(i,j) generated form pseudo-random sequences $M_{2}\left(i,j\right)$ and the DNA encoding rules. The random decoding sequence is described as:

(9) $$De\_M\left(i,j\right)=floor\left(\;\mathrm{mod}\;\left(M_{2}\left(i,j\right),8\right)\right)+1.$$

**Step 7:** Perform the DNA Ex-OR operations and then convert them into the decimal matrices $R_{4}\left(i,j\right)$, $G_{4}\left(i,j\right)$ and $B_{4}\left(i,j\right)$ with the size of M×N. Perform scrambling operations on the decimal three matrices with three pseudo-random sequences $M_{1}\left(i,j\right)$, $M_{2}\left(i,j\right)$ and $M_{3}\left(i,j\right)$, respectively and obtained decimal three matrices $R_{5}\left(i,j\right)$, $G_{5}\left(i,j\right)$ and $B_{5}\left(i,j\right)$ with the size of M×N.

**Step 8:** Perform chaos encryption algorithm on the decimal three matrices $R_{5}\left(i,j\right)$, $G_{5}\left(i,j\right)$ and $B_{5}\left(i,j\right)$ and combine them into an encrypted image.

## 4. Experiment Results and Performance Analysis

**Figure 5:**
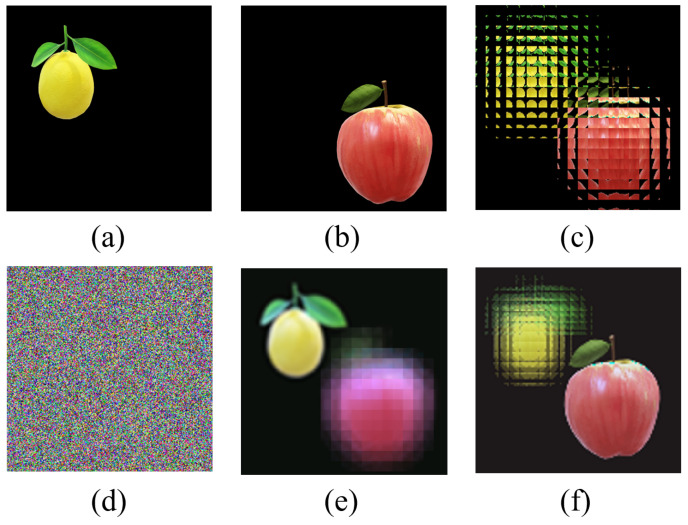
The experiment results of multi-image encryption scheme (240×240). (**a**) Original image “lemon”. (**b**) Original image “apple”. (**c**) EIA of image “lemon” and “apple”. (**d**) Encrypted image. (**e**) Reconstructed image (d = 15 mm). (**f**) Reconstructed image (d = 6 mm).

In this section, the EIA is calculated by the CII algorithm from two images, “lemon” and “apple”. Figure 5 shows the experiment results of the multi-image encryption scheme we proposed. Figure 5a,b shows the original images “lemon” and “apple” with a size of 240×240, and Figure 5c shows the EIA of original images “lemon” and “apple” generated by the CII algorithm. Figure 5d shows the encrypted image using the DNA-chaos algorithm. Figure 5e,f shows images reconstructed by the CIIR algorithm, and the reconstruction depths are 15 mm and 6 mm, respectively.

**Figure 6:**
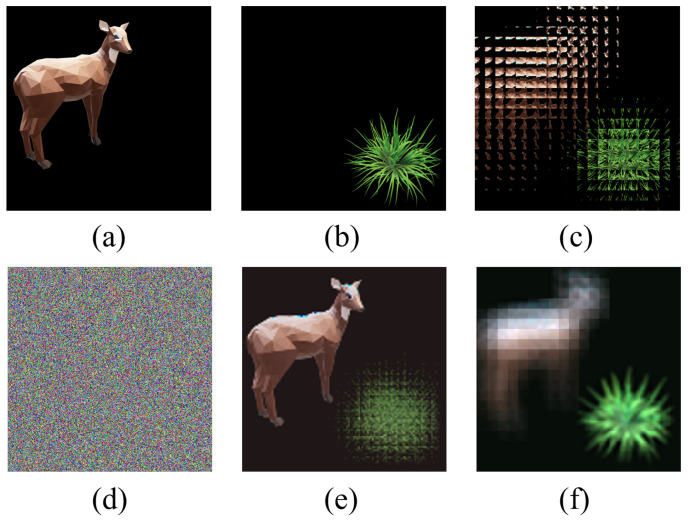
The experiment results of multi-image encryption scheme (360×360). (**a**) Original image “sheep”. (**b**) Original image “grass”. (**c**) EIA of image “sheep” and “grass”. (**d**) Encrypted image. (**e**) Reconstructed image (d = 15 mm). (**f**) Reconstructed image (d = 6 mm).

**Figure 7:**
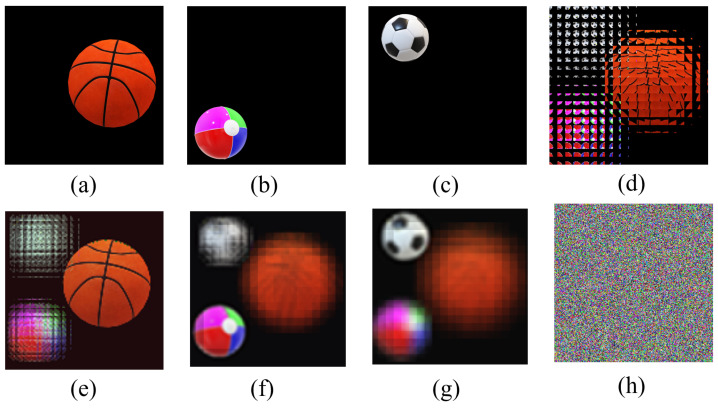
The experiment results with three original images of multi-image encryption scheme (240×240). (**a**) Original image “basketball”. (**b**) Original image “ball”. (**c**) Original image “football”. (**d**) EIA of image “basketball”, “ball” and “football”. (**e**) Reconstructed image (d = 6 mm). (**f**) Reconstructed image (d = 15 mm). (**g**) Reconstructed image (d = 21 mm). (**h**) Encrypted image.

From Figure 5, we can qualitatively see that the multi-image encryption scheme we proposed has an excellent encryption and decryption performance. In order to illustrate that the multi-image encryption scheme we proposed can be applied to different scenarios, we select original images of different sizes and numbers for testing. The experimental results are shown in Figure 6 and Figure 7.

In Figure 6, two original images with a size of 360×360 as the encryption images are different from Figure 5 with a size of 240×240. From Figure 6, we can also qualitatively see that the multi-image encryption scheme we proposed has an excellent encryption and decryption performance.

In Figure 7, three original images with a size of (240×240) are the encryption images. For obvious comparison, we put the encrypted image in the last position. From Figure 7h, we cannot observe any information about the original images. As can be seen from the first three columns in Figure 7, the reconstructed image can clearly restore the information of the original images.

The experimental results in Figure 5, Figure 6 and Figure 7 fully indicate that the multi-image encryption scheme we proposed can be applied to different scenarios, such as original images of different sizes (240×240 in Figure 5 and 360×360 in Figure 6) and different numbers (three original images in Figure 7). Following this section, we will analyze the multi-image encryption scheme we proposed quantitatively by taking two original images as examples.

### 4.1. Key Security Analysis

**Figure 8:**
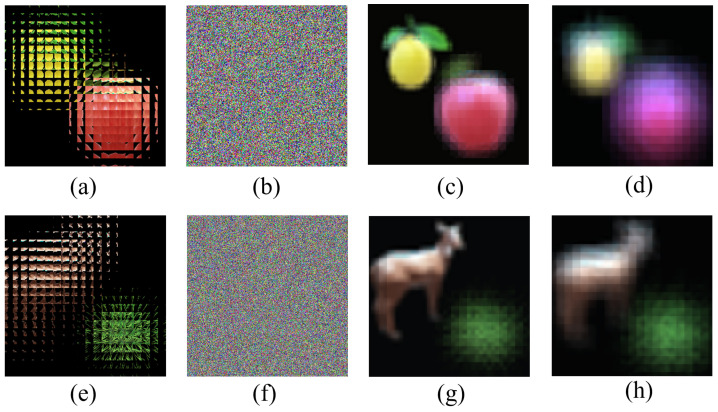
The results of key security analysis. (**a**) The decrypted image of Figure 5d using right key. (**b**) The decrypted image of Figure 5d using wrong key. (**c**) The reconstructed image of Figure 7a with wrong depths (d = 18 mm). (**d**) The reconstructed image of Figure 7a with wrong depths (d = 24 mm). (**e**) The decrypted image of Figure 6d using right key. (**f**) The decrypted image of Figure 6d using wrong key. (**g**) The reconstructed image of Figure 7e with wrong depths (d = 18 mm). (**h**) The reconstructed image of Figure 7e with wrong depths (d = 24 mm).

The encryption scheme must consider the security of the key, that is, the original image cannot be decrypted with the wrong key. Figure 8 shows the results of the key security analysis of our proposed multi-image encryption scheme.

Figure 8 shows the results of key security analysis; the encrypted images corresponding to the first and second columns are Figure 5d and Figure 6d respectively. Figure 8a,e shows the decrypted image with the right key and Figure 8b,f shows the decrypted image with the wrong key; we cannot obtain any useful information about the original image. Figure 8c,d,g,h separately shows the reconstructed image with wrong depths; when the reconstruction depth is wrong, we cannot obtain a clear image relative to the correct reconstruction depth, such as in Figure 5d,h. This shows that the multi-image encryption scheme we proposed has high key security.

### 4.2. Statistical Analysis

**Figure 9:**
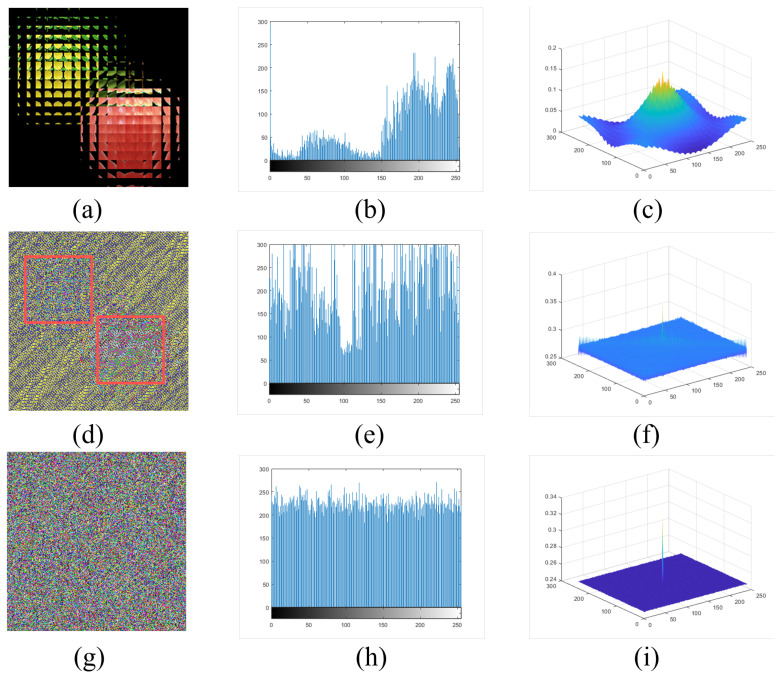
The results of statistical analysis. (**a**) The EIA of two original images. (**d**) The encrypted image only by DNA. (**g**) The encrypted image by DNA-chaos. (**b**,**e**,**h**) represent histogram (R channel) of (**a**,**d**,**g**) respectively. (**c**,**f**,**i**) show autocorrelation results (R channel) of (**a**,**d**,**g**) respectively.

In order to quantitatively illustrate the performance of our proposed multi-image encryption scheme, we performed a statistical analysis of the experimental results, which is shown in Figure 9.

Figure 9a is the EIA of two original images, “lemon” and “apple”, and Figure 9d shows the encrypted image only by DNA algorithm. We can see the outline of two saliency objects in the EIA clearly from the result. Figure 9g represents the image encrypted by the DNA-chaos algorithm, and we cannot see any information about the original image. Figure 9b,e,h represents a histogram (R channel) of Figure 9a,d,g separately. The results of the histogram indicate that the distribution of the image encrypted by DNA-chaos is very flat. Figure 9c,f,i represents the autocorrelation (R channel) of Figure 9a,d,g separately, and we can also find that the autocorrelation is very weak for the image encrypted by DNA-chaos. So the multi-image encryption scheme we proposed has an excellent performance according to the statistical analysis results.

In addition, we calculated the entropy value of Figure 9a,d,g from the RGB channel separately, then took the average of them, and the results are 3.2886, 7.4900 and 7.6277. The entropy value of the encrypted image is significantly larger than the entropy value of the EIA image, which shows that the encryption scheme we proposed performs well.

### 4.3. Robustness Analysis

**Figure 10:**
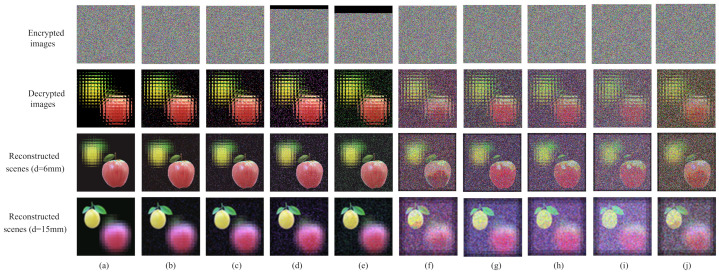
The results of robustness analysis. (**a**) No noise. (**b**) Salt & Pepper noise (0.01). (**c**) Salt & Pepper noise (0.02). (**d**) Clip attack (6.25%). (**e**) Clip attack (12.5%). (**f**) Speckle noise (0.01). (**g**) Speckle noise (0.02). (**h**) Gaussian noise (0.01). (**i**) Gaussian noise (0.02). (**j**) Possion noise.

In order to verify the reliability of the multi-image encryption scheme we proposed in the noisy environment, we designed simulation experiments to analyze the robustness. We simulated Gaussian noise, Speckle noise, Poisson noise, Salt and Pepper noise and Clip attack channel environments to test the robustness of the scheme. The simulation experiment results are shown in Figure 10.

From Figure 10, we can intuitively see that the multi-image encryption scheme we proposed can reconstruct the original scene correctly in a variety of noise environments.

In order to qualitatively illustrate the visual quality the recovered scene, we use the peak signal-to-noise ratio (PSNR) image quality evaluation index that is widely recognized by researchers. The PSNR value of an image can be obtained by the formula [39]:

(10) $$PSNR=10\log_{10}\left(\frac{255^{2}}{MSE\left(E,E^{\prime }\right)}\right),$$

(11) $$MSE=\frac{1}{MN}\sum_{j=0}^{M-1}\sum_{j=0}^{N-1}{\left(E-E^{\prime }\right)}^{2},$$

where *M* and *N* indicate the width and height of the image, respectively, *E* is the original scenes, and $E^{\prime }$ is the recovered scenes.

The PSNR values of the decrypted images with the the Gaussian noise, Speckle noise, Poisson noise, Salt and Pepper noise and Clip attack channel environments are shown in the Table 3.

It can be clearly seen from the Table 3 that the multi-image encryption scheme we proposed has a strong robustness in various noise environments.

There are two indexes to qualitatively illustrate the key sensitivity and plaintext sensitivity encryption scheme, namely number of pixels change rate (NPCR) and unified average changing intensity (UACI) [52]. NPCR indicates the number of pixels that change between two images and UACI represents the average number of changes in intensity between two images. The calculation formula of NPCR and UACI is as follows:

(12) $$\mathrm{NPCR}=\frac{\sum_{i,j}D\left(i,j\right)}{W\times H}\times 100\%$$

(13) $$\mathrm{UACI}=\frac{1}{W\times H}\left[\sum \frac{\left|C_{1}\left(i,j\right)-C_{2}\left(i,j\right)\right|}{255}\right]\times 100,\%$$

where D(i,j) is defined as:

(14) $$D\left(i,j\right)=\left\{\begin{array}{c} 1,C_{1}\left(i,j\right)\neq C_{2}\left(i,j\right)\\ 0,\;\mathrm{otherwise},\; \end{array}\right.$$

where *W* and *H* denote the width and height of the image. C1 and C2 are two images.

That the plaintext images are sensitive is a basic requirement of a good image cryptosystem. Only those image cryptosystems with plaintext sensitivity can resist the chosen/known plaintext attacks. For any given key, if the plain image is changed slightly, its encrypted image will be changed dramatically, and this image cryptosystem is plaintext sensitive.

Key sensitivity analysis includes three aspects: (1) When encrypting a plaintext image, if the key changes slightly, the encryption system will produce two completely different encrypted images, which means that the key is sensitive in the encryption process. Meanwhile, such a key is an effective encryption key; (2) When encrypting a plaintext image, if the key changes slightly, the encryption system will produce two completely different encrypted images, which means that the key is sensitive in the encryption process. Meanwhile, such a key is an effective encryption key; (3) When decrypting a cipher image using an illegal key, if the key changes slightly, the decryption system will produce two completely different images, both totally different from the original plaintext image. This means that the illegal key is sensitive in the decryption process. Such an illegal key is effective.

We use NPCR and UACI to qualitatively illustrate the key sensitivity and plaintext sensitivity of the multi-image encryption scheme we proposed. We still use Figure 5c as our test image; the results are shown in Table 4.

The theoretical value of NPCR is 99.6094% and UACI is 33.4635%. From the Table 4, we can find that the NPCR and UACI are very close to the theoretical value, Which indicates the multi-image encryption scheme we proposed has excellent key sensitivity and plaintext sensitivity.

### 4.4. Time Analysis

**Figure 11:**
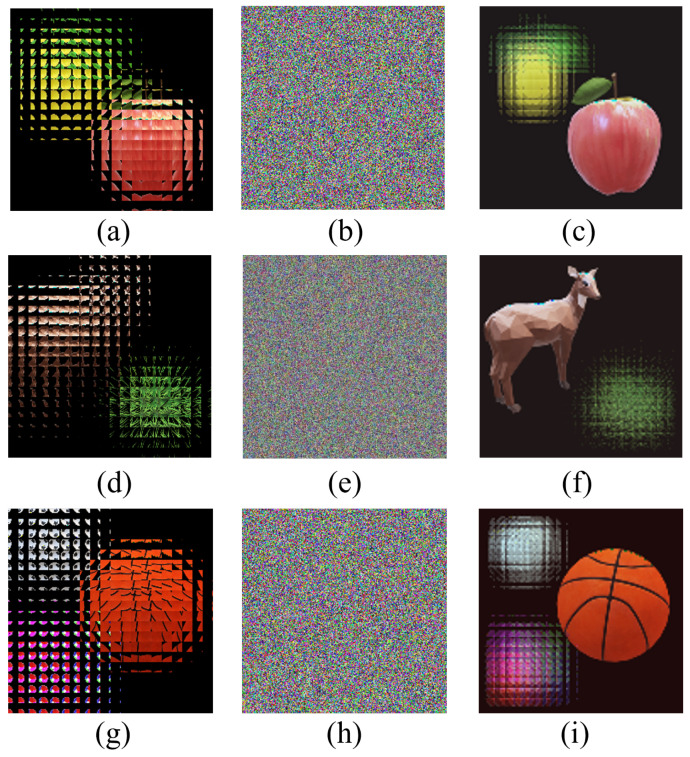
Encryption and decryption analysis of different images. (**a**) The EIA of two input images (240×240). (**d**) The EIA of two input images (360×360). (**g**) The EIA of three input images (240×240). (**b**,**e**,**h**) represent encrypted image of (**a**,**d**,**g**) separately. (**c**,**f**,**i**) show decryption results of (**a**,**d**,**g**) separately.

Encryption time is very important for an encryption scheme. An image encryption scheme with good performance should use as little time as possible in the process of image encryption and decryption. In this section, we select different sizes of images and more than two images as input to test the multi-image encryption scheme we proposed. The results of the encryption and decryption analysis of different images are shown in Figure 11.

Figure 11a is the EIA of two input images with the size of 240×240, Figure 11d is the EIA of two input images with the size of 360×360, and Figure 11g is the EIA of three input images with the size of 240×240. We analysis the encryption and decryption time of these different images. For Figure 11a, the encryption and decryption time is 3.1225 s, Figure 11d is 4.3257 s and Figure 11d is 4.6328 s.

## 5. Conclusions

In conclusion, we apply the CII algorithm to achieve multi-image encryption, which significantly improves the efficiency of image encryption, meanwhile combining the chaos algorithm to address the outline effect caused by the DNA encryption algorithm. In the proposed multi-image encryption scheme, the different depth distances of multiple images can also be used as keys, which can improve the security of the encryption method significantly. We also analyze the robustness of this scheme against a variety of attacks. The experiment results confirm the excellent performance of our proposed multi-image encryption scheme.

## Figures and Tables

**Table 1:** DNA coding rules.

Rule	One	Two	Three	Four	Five	Six	Seven	Eight
A	00	00	01	01	10	10	00	11
C	01	10	00	11	00	11	01	10
G	10	01	11	00	11	00	10	01
T	11	11	10	10	01	01	00	00

**Table 2:** DNA addition, subtraction and XOR operations.

+	A	T	C	G	−	A	T	C	G	⊕	A	T	C	G
G	G	A	T	C	G	G	C	T	A	G	G	C	T	A
C	C	G	A	T	C	C	T	A	G	C	C	G	A	T
T	T	C	G	A	T	T	A	G	C	T	T	A	G	C
A	A	T	C	G	A	A	G	C	T	A	A	T	C	G

**Table 3:** PSNRs of Decrypted Scenes Against Attacks With Different Schemes.

Attacks	R (dB)	G (dB)	B (dB)
Gaussian (0.01)	31.5444	33.0175	34.5347
Gaussian (0.02)	31.3144	32.8944	34.8444
Speckle (0.01)	31.7404	33.2693	34.7687
Speckle (0.02)	31.7186	33.2382	34.4183
Possion	31.7075	33.0537	34.3427
Salt & Pepper (0.01)	43.6942	47.6408	50.1849
Salt & Pepper (0.02)	40.8340	43.9806	46.3331
Clip (6.25%)	48.6002	43.7822	53.1990
Clip (12.5%)	38.4986	41.1150	44.3709

**Table 4:** The analysis of key sensitivity and plaintext sensitivity using NPCR and UACI.

Index	NPCR (%)	UACI (%)
**Plaintext sensitivity**	99.6091	33.4591
**Encryption process**	99.6100	33.4603
**Decryption process (legal)**	99.6064	28.6356
**Decryption process (illegal)**	99.6085	33.4673
**Theoretical value**	**99.6094**	**33.4635**

## Data Availability

Not applicable.
